# 25-OH Vitamin D blood serum linkage with VDR gene polymorphism (rs2228570) in thyroid pathology patients in the West-Ukrainian population

**DOI:** 10.25122/jml-2021-0101

**Published:** 2021

**Authors:** Iryna Ivanivna Kamyshna, Larysa Borysivna Pavlovych, Igor Volodymyrovych Malyk, Aleksandr Mychailovich Kamyshnyi

**Affiliations:** 1. Department of Medical Rehabilitation, I. Horbachevsky Ternopil National Medical University, Ternopil, Ukraine; 2. Department of Clinical Immunology, Allergology and Endocrinology, HSEEU Bukovinian State Medical University, Chernivtsi, Ukraine; 3. Department of the System Analysis and Insurance and Financial Mathematics, Yuriy Fedkovych Chernivtsi National University, Chernivtsi, Ukraine; 4. Department of Microbiology, Virology, and Immunology, I. Horbachevsky Ternopil National Medical University, Ternopil, Ukraine

**Keywords:** 25-OH vitamin D, VDR, autoimmune thyroiditis, hypothyroidism

## Abstract

Vitamin D is known to alter immune regulation. It binds to the vitamin D receptors (VDR) expressed on T lymphocytes and macrophages. In individuals with Hashimoto’s thyroiditis, serum vitamin D levels were found to be lower compared to healthy controls. The study’s objective was to investigate the association between VDR gene polymorphism (rs2228570) with blood serum levels of 25-OH vitamin D in patients with thyroid pathology from western Ukraine. The study involved a total of 153 patients with various forms of thyroid pathology. 25-OH vitamin D levels in the serum of the patients and healthy individuals were quantified with ELISA using the 25-OH vitamin D Total (Vit D-Direct) Test System ELISA Kit (Monobind Inc.®, United States, Product Code: 9425-300) on the EIA Reader Sirio S (Seac, Italy). Genotyping of the VDR (rs2228570) gene polymorphism was performed using TaqMan probes and TaqMan Genotyping Master Mix (4371355) on CFX96™Real-Time PCR Detection System (Bio-Rad Laboratories, Inc., USA). Polymerase chain reaction (PCR) for TaqMan genotyping was carried out according to the kit instructions (Applied Biosystems, USA). Our research identified that that genotype variants of VDR rs2228570 are not risk factors for reduced serum 25-OH vitamin D or vitamin D deficiency in patients with various forms of thyroid pathology patients in the West-Ukrainian population. Vitamin D levels were significantly lower in the carriers of AA and AG genotypes with hypothyroidism caused by autoimmune thyroiditis. In AA genotype carriers with postoperative hypothyroidism, 25-OH vitamin D levels were significantly lower compared to AA genotype carriers with autoimmune thyroiditis.

## Introduction

Hashimoto’s thyroiditis (HT), an autoimmune thyroid disorder (AITD), is one of the leading causes of hypothyroidism, affecting up to 5% of the population. HT is known as lymphocytic thyroiditis and presents with diffuse infiltration of chronic lymphocytic cells and elevated serum thyroid antibodies [1]. Current evidence suggests that HT is a multifactorial disorder, meaning it is determined by both genetic and environmental risk factors [2]. For instance, a polymorphism in immunomodulatory genes, such as forkhead box P3 (FOXP3), cytotoxic T-lymphocyte-associated protein-4 (CTLA-4), and human leukocyte antigen (HLA) family, were linked to the susceptibility to HT [3]. However, the specific contribution of these and other genetic factors and environmental effects to the development of HT are not yet fully understood.

Vitamin D, besides its well-recognized role in calcium metabolism, also affects immune regulation. It binds to vitamin D receptors (VDR) expressed on T lymphocytes and macrophages [4]. In individuals with HT, the serum vitamin D levels were found to be lower compared to healthy controls [5]. This inverse association suggests that vitamin D deficiency might be among the environmental factors contributing to HT. Moreover, when evaluating this risk factor, its interaction with VDR expression should be taken into account.

The active form of vitamin D, 1,25(OH)2D, is a vital immunomodulator since it triggers innate and adaptive immune responses through binding to VDR, which is a ligand inducible transcription factor expressed on many immune cells and a member of the family of trans-acting transcriptional factors. The gene encoding VDR on chromosome 12q13.11 spans approximately 75 kb and contains 14 exons. Among more than sixty single nucleotide polymorphisms (SNPs) identified in the VDR gene, some, including rs731236, rs1544410, rs2228570, and rs7975232, have been linked to AITD risk [5–7]. While these SNPs seem to modify vitamin D action, their contribution to AITD is not yet fully defined.

The association of the four common VDR SNPs, namely TaqI (rs731236, exon 9, +65058 T > C), ApaI (rs7975232, intron 8, +64978 C > A), FokI (rs2228570, exon 2, +30920 C > T), and BsmI (rs1544410, intron 8, + 63980 G > A) with various human traits have been intensively studied. These polymorphisms were linked to the risk of several autoimmune disorders, including rheumatoid arthritis, systemic lupus erythematosus, inflammatory bowel disease, diabetes mellitus, and AITD, including HT [5, 8]. However, these studies did not analyze the relationship between VDR polymorphisms and vitamin D levels. Therefore, the mechanisms and effects of the interaction of vitamin D and VDR in patients with HT remain unclear. In previous studies, we demonstrated that autoimmune thyroiditis (AIT) and hypothyroidism could affect the transcription of the genes involved in neurogenesis, nerve impulse transmission, and cell cycle regulation [8, 10–13]. These changes in gene expression may play a role in the development of neurological complications associated with thyroid pathology. This study aimed to examine the association between a VDR polymorphism (rs2228570) with blood serum levels of 25-hydroxy cholecalciferol in patients with thyroid pathology from western Ukraine.

## Material and Methods 

Study participants (n=153) were divided into three groups depending on the form of thyroid pathology. Group 1 (postoperative hypothyroidism – PO, n=16) comprised patients with postoperative hypothyroidism; group 2 (AIT with hypothyroidism, n=65) comprised patients with hypothyroidism caused by autoimmune thyroiditis; and group 3 (AIT, n=72) comprised patients with AIT accompanied by elevated serum antibodies, anti-thyroglobulin, and anti-thyroid peroxidase. The control group (n=25) comprised individuals recruited randomly without matching for age or sex. The demographic, clinical, and biochemical characteristics of the participants are presented in Table 1.

**Table 1.** Demographic, clinical, and biochemical characteristics of study participants.

	**Control group (n=25)**	**Patients with postoperative hypothyroidism (PO, n=16)**	**Patients with AIT-induced hypothyroidism (AIT with hypothyroidism, n=65)**	**Patients with AIT and elevated anti-Tg and anti-TPO antibodies (AIT, n=72)**
**Age (years)**	46.08±14.58	47.30±12.27	46.72±15.49	45.02±13.65
**fT4 (pmol/L)**	8.91±0.97	3.44±0.31	4.13±0.52	8.51±0.82
**TSH (mIU/mL)**	2.67±0.52	8.61±0.84	7.09±0.50	2.38±0.62
**anti-TPO (IU/mL)**	34.04±3.70	36.13±2.78	380.62±73.42	330.36±50.23
**anti-TG (IU/mL)**	15.32±1.97	15.50±1.90	32.97±4.27	36.38±7.70
**Current dose of L-thyroxine (μg/day)**	None	110.95±5.25	88.46±1.55	None
**25-OH Vitamin D**	39.2±6.58	20.69±3.09 (p<0.001)	19.08±3.144 (p<0.001)	21.48±2.83 (p<0.001)

Data are presented as mean ± standard deviation. * p-value between control and study groups.

In the individuals of the study group, hypothyroidism was diagnosed following the 2012 Guidelines from the American Association of Clinical Endocrinologists. The AIT diagnosis was based on clinical signs and symptoms, reduced echogenicity on the thyroid sonogram, and a positive test for circulating antibodies to thyroid antigens, anti-thyroid peroxidase, and anti-thyroglobulin.

Blood samples from the individuals of both study and control groups were collected in the morning (8 to 10 AM) after an overnight fast. Levels of thyroxine (fT4, normal range 6.0–13.0 pmol/L for males and 7.0–13.5 pmol/L for females), thyroid-stimulating hormone (TSH, normal range 0.3–4.0 mIU/mL), anti-thyroid peroxidase (anti-TPO, normal range 0–30 IU/mL) and anti-thyroglobulin (anti-TG, normal range 0–65 IU/mL), were determined on a STAT FAX303/Plus analyzer (Awareness Technology Inc, USA).

Study and control groups had the following exclusion criteria: age <18 years, presence of malignancy, inflammation caused by rheumatic diseases or acute/chronic infection, diabetes mellitus, vascular, chronic hepatic or renal diseases, and pregnancy. Individuals taking any medications that could interfere with thyroid function were also excluded from the study.

### 25-OH vitamin D quantification using enzyme-linked immunosorbent assay (ELISA)

25-OH vitamin D levels in the serum of the patients and healthy individuals were quantified with ELISA using the 25-OH Vitamin D Total (Vit D-Direct) Test System ELISA Kit (Monobind Inc.®, United States, Product Code: 9425-300) on EIA Reader Sirio S (Seac, Italy).

### Detection of VDR rs2228570 polymorphism DNA isolation

Venous blood was collected in a sterile Vacutainer and stabilized with K2EDTA. Total DNA was isolated from the blood using PREP-RAPID-GENETICS DNA Extraction Kit (DNA-TECHNOLOGY, Russian Federation) following the manufacturer’s protocol.

### DNA amplification and genotyping

**Figure 1.**
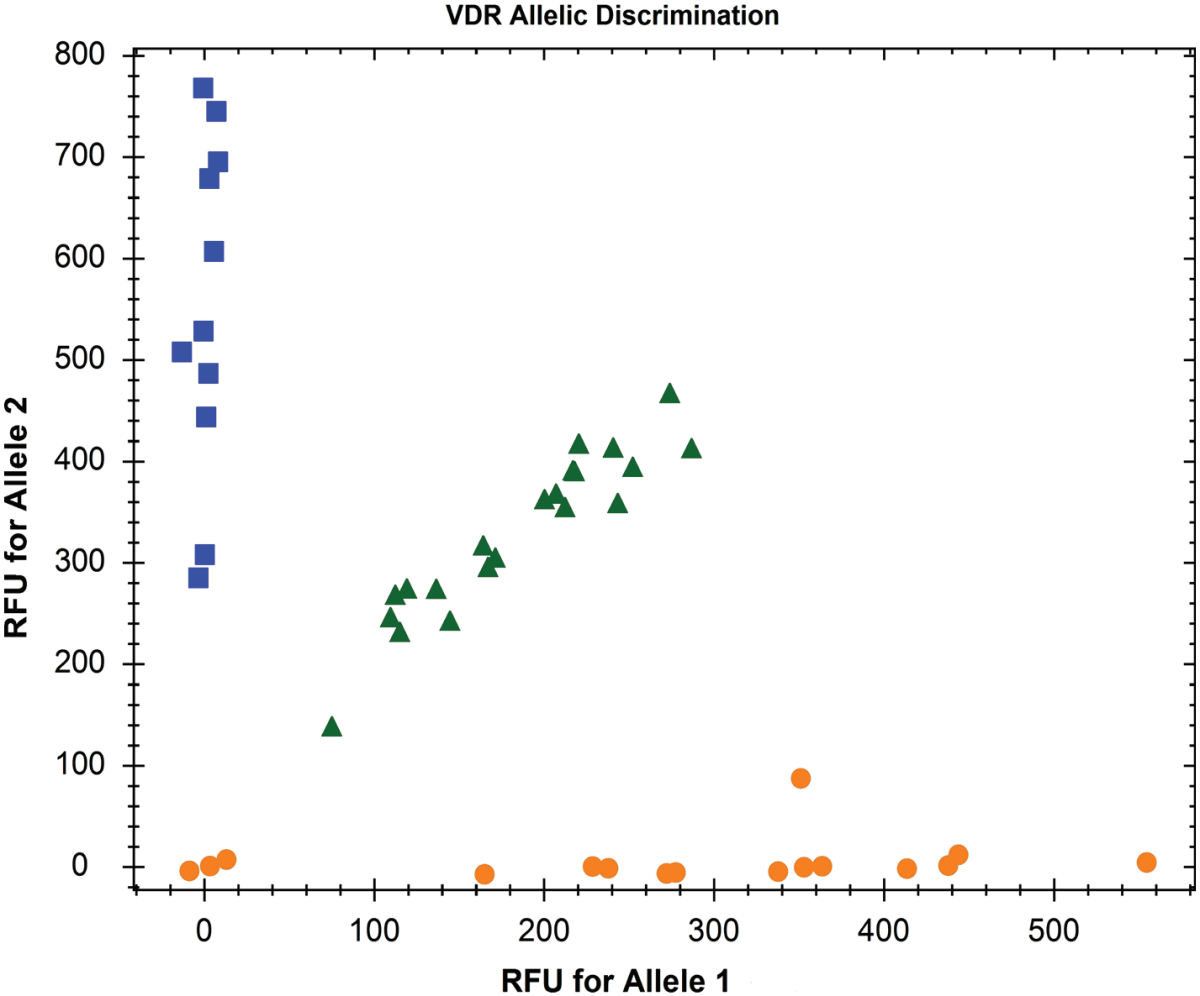
Allelic discrimination analysis is based on the magnitude of relative fluorescence units (RFU).

The samples were genotyped using TaqMan probes and TaqMan Genotyping Master Mix (4371355; Applied Biosystems, USA) on CFX96™Real-Time PCR Detection System (Bio-Rad Laboratories, Inc., USA). Polymerase chain reaction (PCR) according to manufacturer’s protocol. TaqMan Genotyping Master Mix contains DNA polymerase AmpliTaq Gold®, dNTPs, reference dye ROX™, and buffer ingredients. TaqMan probes are target-specific oligonucleotides with reporter dyes attached to the 5’ end of each probe: (VIC® dye on the 5’ end of the Allele 1 probe and 6FAM ™ dye on the 5’ end of the Allele 2 probe), and a non-fluorescent quencher (NFQ) the 3’ end of the probe. Genomic DNA was amplified in a 10 μL reaction mix containing genomic DNA, forward and reverse primers, fluorescent probes, and TaqMan Genotyping Master Mix. Genotyping of the samples performed on the CFX-Manager ™ software using the method of allele discrimination based on the magnitude of relative fluorescence units (RFU) (Figure 1).

### Statistical analysis

The difference between groups was determined using student’s t-test, ANOVA, Pearson’s χ2 test, odds ratio test, relative odds ratio test, and equality 0 correlation test. Binary logistic regression was used to calculate the odds ratio and 95% confidence interval (CI). P values <0.05 were considered statistically significant.

## Results

We analyzed allele and genotype frequencies of rs2228570 in patients with thyroid pathology and the control group. The relative frequency of these gene variants did not differ between the groups (Table 2).

**Table 2.** Distribution of the rs2228570 genotype and allelic frequencies in the surveyed population.

**rs2228570** **n=178** **(%)**	**Study group,** **n=153** **(86.96%)**	**Control group,** **n=25** **(14.04%)**	**OR** **[95% CI]**	**χ^2^ (p)**
**AA**	59 (38.56%)	9 (36%)	1.115 [0.43; 306]	**χ^2^<1.0** **p>0.05**
**AG**	64 (41.83%)	9 (36%)	1.277 [0.494; 3.497]	χ^2^<1.0 p>0.05
**GG**	30 (19.61%)	7 (28%)	0.629 [0.224; 1.948]	χ^2^ <1.0 p>0.05
**χ^2^ P**	χ^2^=13.216, p<0.001	χ^2^=0.32, p=0.8521		
**A allele**	182 (59.48%)	27 (54%)	1.249 [0.652; 2.38]	χ^2^ <1.0 p>0.05
**G allele**	124 (40.52%)	23 (46%)		

OR - odds ratio; n - total number

The odds ratio (OR) test indicates that the distribution of AA, AG and GG genotypes do not differ between the study and control groups. The AG genotype was predominant in the surveyed population: 41.83% and 36% in the study and control groups, respectively. The homozygous AA genotype was the second most common: 38.56% and 36%, respectively. The GG genotype was the least common: 19.61% in the study group and 28% in the control group (p>0.05).

The frequency of both alleles did not differ between the groups: among the thyroid pathology patients, 59.48% carried the A allele vs. 54% in the control group (p>0.05); the allelic frequencies for the G allele were 40.52% and 46% in the study and control group, respectively (p>0.05).

Distribution of rs2228570 variants in the patients depending on the type of thyroid pathology showed no significant difference in the relative frequency of this polymorphism among the patients of PO, AIT with hypothyroidism, and AIT groups (Table 3).

**Table 3.** Distribution of the rs2228570 genotypes in the study group depending on the type of thyroid pathology and in the control group.

**rs6265 genotype**	**Control group,** **n=25** **(14.04%)**	**Study Group.** **n=153** **(85.96%)**	**Group 1, PO** **n=16 (10.46%)**	**Group 2,** **AIT with hypothyroidism** **n=65** **(42.48%)**	**Group 3,** **AIT** **n=72** **(47.06%)**	**χ^2^** **p**	**Total** **n=178** **(%)**	**OR** **[95% CI]**
**AA**	9 (36%)	59 (38.56%)	7 (43.75%)	20 (30.77%)	32 (44.44%)	χ^2^=2.9695, p=0.3964	68 (30.2%)	0.706 [0.24; 1.96]
**AG**	9 (36%)	64 (41.83%)	6 (37.5%)	33 (50.77%)	25 (34.72%)	χ^2^=4.08, p=0.2533	73 (41%)	1.06 [0.35; 2.98]
**GG**	7 (28%)	30 (19.61%)	3 (18.75%)	12 (18.46%)	15 (20.83%)	χ^2^=1.04, p=0.7	37 (20.8%)	1.47 [9.44; 4.6]
**χ^2^** **P**	χ^2^=0.32, p=0.8521	χ^2^=13.216, p<0.001	χ^2^=1.65, p=0.4437	χ^2^=10.369, p<0.001	χ^2^=6.08, p=0.048			

All patients suffering from hypothyroidism exhibited significantly reduced serum 25-OH vitamin D levels compared to the control group: PO - 1.89 times, AIT without hypothyroidism - 1.82 times, and AIT - 2.05 times (Table 1).

Analysis of serum 25-OH vitamin D levels depending on the study group and genotype showed that in the control group, 25-OH Vitamin D levels were the lowest in carriers of the AA genotype, by 17.1% compared with carriers of the GG genotype (Table 4).

In all groups of patients, 25-OH vitamin D levels were significantly lower regardless of genotypes compared to the control group (Table 4). For instance, in the study group, there was a significant decrease in 25-OH Vitamin D levels in the carriers of AA and AG genotypes by 43.5% and 47.4%, respectively, compared with the control group. Similarly, the carriers of the GG genotype of the study group had 53.9% reduced levels of 25-OH Vitamin D, compared to the control group (p<0.001).

In patients with postoperative hypothyroidism, the level of 25-OH vitamin D was significantly lower in the carriers of all genotypes: AA, AG, and GG (by 46%, 45%, and 49.3% respectively by compared to the control group (p<0.001). At the same time, in the group of patients with postoperative hypothyroidism, there were no significant differences in the levels of 25-OH vitamin D regardless of whether they were carriers of the genotype AA, AG, or GG.

In patients with AIT-induced hypothyroidism, the levels of 25-OH vitamin D were significantly lower in the carriers of AA, AG, and GG genotypes, respectively 1.96, 2, and 2.28 times, compared to the control group (p<0.001).

In the group of patients with AIT, 25-OH vitamin D levels were also significantly lower compared to the control group (in carriers of the AA genotype: 39.4%; AG: 44.5%; and GG: 52.8%) (Table 4). Comparative analysis of the 25-OH Vitamin D levels between patients with AIT-induced hypothyroidism and AIT without hypothyroidism shows that they were significantly lower in the patients with AIT-induced hypothyroidism carriers of the AA and AG genotypes.For instance, 25-OH vitamin D in the carriers of AA genotype with AIT-induced hypothyroidism was 15.8% lower compared to the AA genotype carriers with AIT. Additionally, in the AA genotype carriers with postoperative hypothyroidism, 25-OH vitamin D levels were also significantly reduced (by 18.8%) compared to the AA genotype carriers with AIT. Thus, the carriers of the AA genotype with hypothyroidism, regardless of its cause (autoimmune or postoperative), had significantly lower 25-OH vitamin D levels compared to the carriers of the AA genotype without hypothyroidism.

**Table 4.** 25-OH vitamin D levels in groups of patients with different thyroid pathology depending on the rs2228570 genotype.

**rs2228570 (M±m)**						
**rs2228570 genotype**	**AA**	**AG**	**GG**	**P5**	**P6**	**P7**
Control group, n=25	36±5.05 (9)	39.11±6.92 (9)	43.43±6.27 (7)	0.29	0.026	0.21
Study group, n=153	20.35±3.28 (59)	20.56±3.29 (64)	20.03±2.74 (30)	0.73	0.625	0.416
P1	<0.001	<0.001	<0.001			
**PO n=16**	19.43±2.07 (7)	21.5±2.88 (6)	22±5.29 (3)	0.17	0.49	0.889
P1	<0.001	<0.001	<0.0035			
P2	0.322	0.175	0.431			
**AIT with** **hypothyroidism n=65**	18.35±3.18 (20)	19.55±3.27 (33)	19±2.49 (12)	0.197	0.5254	0.5568
P1	<0.001	<0.001	<0.001			
P3	0.0003	0.0138	0.121			
**AIT n=72**	21.8±2.86 (32)	21.68±3.08 (25)	20.47±2.17 (15)	0.87	0.083	0.153
P1	<0.001	<0.001	<0.001			
P4	0.0256	0.89	0.667			

P1 – p-value between the control group and study groups; P2 – p-value between the PO and AIT with hypothyroidism groups; P3 – p-value between the AIT and AIT with hypothyroidism groups; P4 – p-value between the PO and AIT groups; P5 – p-value between the AA and AG genotypes; P6 – p-value between the AA and GG genotypes; P7 – p-value between the AG and GG genotypes.

Genotype variants of VDR rs2228570 are not risk factors for reduced serum 25-OH Vitamin D, either vitamin D deficiency or suboptimal provision (Table 5). A correlation analysis between vitamin D levels and the levels of TSH, T4, anti-Tg, and anti-TPO antibodies showed a weak direct relationship (r=0.25) between vitamin D and TSH levels (p<0.001), a weak inverse correlation (r=-0.24) between vitamin D and T4 levels in the blood (p<0.001) and a weak direct relationship (r=0.26) between anti-Tg and vitamin D levels (p=0.0157). In addition, we found a significant direct correlation between the levels of anti-TPO and anti-Tg (p<0.001).

**Table 5.** rs2228570 genotypes as risk factors for reduced serum 25-OH vitamin D levels.

**rs2228570 genotypes**		**RelR**	**OR**	**95% CI RR**	**95% CI OR**	**p**
**Vitamin D <20 ng/mL (Vit D deficiency)**	AA	0.95	0.867	[0.674; 1.343]	[0.294; 2.66]	0.804
	AG	0.64	1.34	[0.778; 1.62]	[0.466; 4.055]	0.631
	GG	0.95	0.82	[0.714; 1.26]	[0.256; 2.82]	0.785
**Vitamin D = 20–30 ng/mL (Suboptimal Vit D availability)**	AA	1.09	1.24	[0.769; 1.548]	[0.45; 3.65]	0.814
	AG	1.09	1.24	[0.769; 1.548]	[0.45; 3.65]	0.814
	GG	0.87	0.54	[0.667; 1.136]	[0.169; 1.858]	0.26

RelR (relative risk) – relative risk; OR (Odds Ratio) – odds ratio; 95% CI RR, OR (confidence interval) – confidence interval of risk (RR), chances (OR).

## Discussion

One of the main directions of biomedical research today is identifying genetic factors and their role in developing multifactorial diseases [14]. Analysis of the genome can be used to detect these hereditary factors [15, 16]; often, mutations contributing to the increased disease risk are single nucleotide polymorphisms [17–19].

In this study, we investigated the correlation of a polymorphism at the rs2228570 locus of the VDR with serum 25-OH vitamin D levels in patients with thyroid gland pathologies in western Ukraine.

Recent evidence shows an association between low vitamin D status and autoimmune thyroid diseases such as Hashimoto’s thyroiditis. In several clinical studies, patients with AITD or HT had a low vitamin D status, indicating the link between vitamin D deficiency and thyroid autoimmunity [20]. Our results show a significant decrease in serum vitamin D levels in patients with postoperative hypothyroidism and AIT-induced hypothyroidism (1.89 and 2.05 times, respectively), compared to the control group. Moreover, in the AIT patients without hypothyroidism, the level of vitamin D was significantly decreased (1.82 times) compared to the control group. An analysis of the correlation between vitamin D levels and the levels of TSH, T4, anti-Tg, and anti-TPO antibodies in the blood show a weak direct relationship (r=0.25) between vitamin D and TSH levels (p<0.001), a weak inverse correlation (r=-0.24) between vitamin D and T4 levels (p<0.001) and a weak direct relationship (r=0.26) between anti-Tg and vitamin D levels (p=0.0157). Additionally, we found a significant direct correlation between anti-TPO and anti-Tg levels (p<0.001).

To date, four polymorphisms of the VDR gene (TaqI (rs731236, alleles T/t), ApaI (rs7975232, alleles A/a), FokI (rs2228570, alleles F/f), and BsmI (rs1544410, alleles B/b) have been linked to the HT risk. However, studies focusing on these polymorphisms have produced inconsistent results. A meta-analysis of 11 case-control studies aimed to establish an association between the four polymorphisms and HT susceptibility [21]. In this meta-analysis, only FokI polymorphism was found to be significantly associated with the risk of HT emergence (F vs. f: OR=1.44, 95% CI=1.09–1.91, p=0.010; FF vs. Ff + ff: OR=1.72, 95% CI=1.09–2.70, p=0.019). Furthermore, subgroup analyses only showed significant effects in the Asian population (F vs. f: OR=1.45, 95% CI=1.07–1.95, p=0.016; FF vs. ff: OR=1.64, 95% CI=1.03–2.59, p=0.036; FF + Ff vs. ff: OR=1.34, 95% CI=1.00–1.80, p=0.047; and FF vs. Ff + ff: OR=1.64, 95% CI=1.03–2.64, p=0.039); no significant effects were present in the Caucasian population. For the rest of the polymorphisms, TaqI, ApaI, and BsmI, there were no significant associations found under any model. This available evidence suggested that only VDR FokI polymorphism is associated with HT risk, and only in Asian, but not in Caucasian, population [21].

In this study, we found that among the groups of patients with different types of thyroid pathology (postoperative hypothyroidism, and AIT without hypothyroidism), there were no significant differences in the relative frequencies of VDR FokI polymorphism.

Comparative analysis of 25-OH vitamin D levels in the AIT patients with and without hypothyroidism shows that it was significantly lower in the carriers of AA and AG genotypes with AIT-induced hypothyroidism. For example, 25-OH vitamin D levels in AA genotype carriers with AIT-induced hypothyroidism were reduced by 15.8% compared to the AA genotype carriers with AIT and no hypothyroidism. Additionally, in AA genotype carriers with postoperative hypothyroidism, 25-OH vitamin D levels were significantly lower (by 18.8%) compared to AA genotype carriers with AIT.

In this study, we found that AA genotype carriers with hypothyroidism, regardless of its cause (autoimmune or postoperative), had significantly lower levels of 25-OH Vitamin D compared to the AIT patients without hypothyroidism. Thus, against the background of thyroid gland pathology, reduced levels of vitamin D exacerbate thyroid insufficiency.

The FokI (rs2228570) polymorphism, affecting the translational initiation codon of VDR, is the only currently known polymorphism of this gene that results in the expression of an altered protein [22]. Two structurally distinct protein isoforms are being produced: F-VDR is 3 amino acid residues shorter than f-VDR. The shortened F-VDR protein variant has been reported to be more effective in vitamin D-induced transactivation [23]. For instance, in transfection experiments, the shortened F-VDR produced higher NF-kB- and NFAT-mediated transcription activity compared to f-VDR. Thus, a FF VDR genotype results in elevated expression of both mRNA and protein IL-12 in human monocytes and dendritic cells compared to the cells with an ff VDR genotype [24]. This suggests that FF genotype carriers may produce a more robust immune response and thus possess an increased risk of developing immune-mediated disorders.

Six out of eight previous studies comparing frequencies of FokI polymorphism in HT patients with controls found a positive association [25]. A meta-analysis by Wang al. concludes that the F allele might be a risk factor for HT susceptibility (OR=1.44, p=0.010) since the incidence of HT was significantly higher in individuals with FF genotype compared to Ff + ff genotype individuals (OR=1.72, p=0.019). However, analyses of subgroups stratified by ethnicity indicated that while the risk of HT in FF genotype individuals was higher in Asian populations (OR=1.64, p=0.039), the same did not hold for Caucasian populations. This inconsistency suggests the impact of other factors, such as different genetic backgrounds and environmental and lifestyle causes such as diet and exposure to sunlight. However, a relatively small Caucasian sample size might have affected the robustness of the analysis.

Results of our study show that the rs2228570 VDR polymorphism is not a risk factor for reduced serum 25-OH vitamin D, either vitamin D deficiency or its suboptimal provision. Studies demonstrated that VDR gene polymorphisms (rs731236, rs1544410, rs2228570, and rs7975232) could alter VDR expression [26]. For instance, the f allele of VDR rs2228570 was associated with higher VDR mRNA copy numbers [27]. Some studies investigated associations between these polymorphisms and various disorders, including autoimmune diseases and HT; however, it is apparent that findings often vary by race and ethnicity. For instance, a significant association between VDR rs2228570 and HT risk was reported in Serbian populations [28], while VDR rs731236 and rs2228570 were significantly associated with HT risk in a Turkish population [29]. At the same time, other studies, including a genome-wide association study, failed to associate these polymorphisms with susceptibility to AITD [5]. A meta-analysis of VDR rs2228570 polymorphism and AITD risk showed a significant association the overall analysis (CT versus CC: OR=0.73, 95% CI: 0.56–0.95, PZ=0.02; TT+CT versus CC: OR=0.71, 95% CI: 0.54–0.93, PZ<0.001; T versus C: OR=0.80, 95% CI: 0.68–0.95, PZ=0.01) [30]. While a further subgroup analysis showed a significant association with HT (T versus C: OR=0.69, 95% CI: 0.50–0.97, PZ=0.03), there was no association with Graves’ disease (GD). A subgroup analysis taking into account ethnicity found a significant association in Asian populations (TT versus CC: OR=0.63, 95% CI: 0.42–0.93, PZ=0.02; TT+CT versus CC: OR=0.65, 95% CI: 0.45–0.95, PZ=0.02; TT versus CT+CC: OR=0.72, 95% CI: 0.58–0.91, PZ=0.005; T versus C: OR=0.72, 95% CI: 0.56–0.92, PZ=0.008), but not in Caucasian populations. These results suggest the effect of other factors, such as the genetic background of different populations; however, they can also be explained by a study design with small sample sizes resulting in low statistical power when detecting low-penetration polymorphisms.

## Conclusion

Vitamin D levels were significantly lower in carriers of the AA and AG genotypes with AIT-induced hypothyroidism. In AA genotype carriers with postoperative hypothyroidism, 25-OH vitamin D levels were significantly lower (by 18.8%) compared to AA genotype carriers with AIT. Variants of the VDR rs2228570 polymorphism are not risk factors for reduced serum 25-OH vitamin D in patients with various forms of thyroid pathology. The question of whether reduced 25-OH vitamin D levels are associated with increased risk of developing AITD requires further investigation, in particular ethnically representative randomized controlled studies to determine the usefulness and safety of vitamin D supplementation as a treatment approach for thyroid diseases.

## Acknowledgments

### Ethical approval

The approval for this study was obtained from the Ethics Committee of the HSEEU Bukovinian State Medical University and Chernivtsi Regional Endocrinology Center, Ukraine (approval ID: 11-07.11.2017).

### Consent to participate

Written informed consent was obtained from the patients.

### Data availability

Further data are available from the corresponding author on reasonable request.

### Conflict of interest

The authors declare that there is no conflict of interest.

## References

[1] Lee H. J., Li C. W., Hammerstad S. S., Stefan M., Tomer Y. (2015). Immunogenetics of autoimmune thyroid diseases: A comprehensive review.. J. Autoimmun..

[2] Hasham A., Tomer Y. (2012). Genetic and epigenetic mechanisms in thyroid autoimmunity.. Immunol. Res..

[3] Ramesh B. G. (2015). Genomics and phenomics of Hashimoto’s thyroiditis in children and adolescents: a prospective study from Southern India.. Annals of translational medicine.

[4] Lithgow H, Florida-James G, Ross M, Duncan G, Leggate M (2021). Exercise acutely increases vitamin D receptor (VDR) expression in T-lymphocytes in vitamin D deficient men, independent of age.. Exp Physiol..

[5] Giovinazzo S., Vicchio T. M., Certo R. (2017). Vitamin D receptor gene polymorphisms/haplotypes and serum 25(OH)D3 levels in Hashimoto’s thyroiditis.. Endocrine..

[6] Long X., Wu W. F., Hu Z. Q., Zhou Z. M. (2016). Association between vitamin D receptor polymorphisms and the risk of Graves’ disease.. Chinese Journal of Gerontology..

[7] Uitterlinden A. G., Fang Y., Van Meurs J. B., Pols H. A., Van Leeuwen J. P. (2004). Genetics and biology of vitamin D receptor polymorphisms.. Gene.

[8] Yu F. (2016). Study and Evaluation the Impact of VDR Variants on the Risk of T2DM in Han Chinese.. Journal of diabetes.

[9] Kamyshna I., Pavlovych L, Maslyanko V., Kamyshnyi A (2021). Analysis of the transcriptional activity of genes of neuropeptides and their receptors and their receptors in the blood of patients with thyroid pathology.. Journal of Medicine and Life..

[10] Bilous II, Korda MM, Krynytska IY, Kamyshnyi AM (2020). Nerve impulse transmission pathway-focused genes expression analysis in patients with primary hypothyroidism and autoimmune thyroiditis.. Endocr Regul..

[11] Bilous I., Pavlovych L., Krynytska I., Marushchak M., Kamyshnyi A (2020). Apoptosis and Cell Cycle Pathway-Focused Genes Expression Analysis in Patients with Different Forms of Thyroid Pathology.. Open Access Macedonian Journal of Medical Sciences..

[12] Bilous I., Pavlovych L, Kamyshnyi A (2021). Primary hypothyroidism and autoimmune thyroiditis alter the transcriptional activity of genes regulating neurogenesis in the blood of patients.. Endocr Regul..

[13] Kamyshna I., Kamyshnyi A (2021). Transcriptional Activity of Neurotrophins Genes and Their Receptors in the Peripheral Blood in Patients with Thyroid Diseases in Bukovinian Population of Ukraine.. Open Access Macedonian Journal of Medical Sciences..

[14] Nosulenko IS, Voskoboynik OY, Berest GG, Safronyuk SL, Kovalenko SI, Kamyshnyi OM, Polishchuk NM, Sinyak RS, Katsev AV (2014). Synthesis and Antimicrobial Activity of 6-Thioxo-6,7-dihydro-2H-[1,2,4]triazino[2,3-c]-quinazolin-2-one Derivatives.. Scientia Pharmaceutica..

[15] Degen A, Krynytska I, Kamyshnyi A (2020). Changes in the transcriptional activity of the entero-insular axis genes in streptozotocin-induced diabetes and after the administration of TNF-α non-selective blockers.. Endocrine Regulations..

[16] Putilin DA, Evchenko SY, Fedoniuk LY, Tokarskyy OS, Kamyshny OM, Migenko LM, Andreychyn SM, Hanberher II, Bezruk TO (2020). The Influence of Metformin to the Transcriptional Activity of the mTOR and FOX3 Genes in Parapancreatic Adipose Tissue of Streptozotocin-Induced Diabetic Rats.. J Med Life..

[17] Lyubomirskaya E.S., Kamyshnyi A.M., Krut Y.Y., Smiianov V.A., Fedoniuk L.Y., Romanyuk L.B., Kravets N.Y., Mochulska O.M. SNPs and transcriptional activity of genes of innate and adaptive immunity at the maternal-fetal interface in woman with preterm labour, associated with preterm premature rupture of membranes.. (2020) Wiadomosci lekarskie (Warsaw, Poland: 1960).

[18] Lyubomirskaya K., Krut Y., Sergeyeva L., Khmil S., Lototska O., Petrenko N., Kamyshnyi A (2020). Preterm premature rupture of membranes: Prediction of risks in women of Zaporizhzhia region of Ukraine.. Polski Merkuriusz Lekarski.

[19] Dzhuryak V., Sydorchuk L., Sydorchuk A., Kamyshnyi O., Kshanovska A., Levytska S., Knut R., Sheremet M., Ivashchuk S., Petrynych O., Kazantseva T., Nikyfor L., Melnychuk L., Sokolenko A., Yarynych Y., Semianiv M., Repchuk Y., Voroniuk K., Sydorchuk R., Sokolenko L., Iftoda O., Kushnir O. (2020). The cytochrome 11B2 aldosterone synthase gene CYP11B2 (RS1799998) polymorphism associates with chronic kidney disease in hypertensive patients.. Biointerface Research in Applied Chemistry..

[20] Kim D (2017). The Role of Vitamin D in Thyroid Diseases.. Int J Mol Sci..

[21] Wang X, Cheng W, Ma Y, Zhu J (2017). Vitamin D receptor gene FokI but not TaqI, ApaI, BsmI polymorphism is associated with Hashimoto's thyroiditis: a meta-analysis.. Sci Rep..

[22] Whitfield G.K. (2001). Functionally relevant polymorphisms in the human nuclear vitamin D receptor gene.. Mol. Cell. Endocrinol..

[23] Colin E. M. (2000). Consequences of vitamin D receptor gene polymorphisms for growth inhibition of cultured human peripheral blood mononuclear cells by 1, 25-dihydroxyvitamin D3.. Clinical endocrinology.

[24] van Etten E. (2007). The vitamin D receptor gene FokI polymorphism: functional impact on the immune system.. Eur. J. Immunol..

[25] Guleryuz B., Akin F., Ata M. T., Dalyanoglu M. M., Turgut S. (2016). Vitamin-D Receptor (VDR) Gene Polymorphisms (TaqI, FokI) in Turkish Patients with Hashimoto’s Thyroiditis: Relationship to the levels of Vit-D and Cytokines. Endocr.. Metab. Immune Disord. Drug Targets.

[26] Uitterlinden A. G., Fang Y., van Meurs J. B. J., Pols H. A. P., van Leeuwen J. P. T. M. (2004). Genetics and biology of vitamin D receptor polymorphisms.. Gene..

[27] Ogunkolade B. W., Boucher B. J., Prahl J. M. (2002). Vitamin D receptor (VDR) mRNA and VDR protein levels in relation to vitamin D status, insulin secretory capacity, and VDR genotype in Bangladeshi Asians.. Diabetes.

[28] Djurovic J., Stojkovic O., Ozdemir O. (2015). Association between FokI, ApaI and TaqI RFLP polymorphisms in VDR gene and Hashimoto’s thyroiditis: preliminary data from female patients in Serbia. International Journal of Immunogenetics..

[29] Yazici D., Yavuz D., Tarcin O., Sancak S., Deyneli O., Akalin S (2013). Vitamin D receptor gene ApaI, TaqI, FokI and BsmI polymorphisms in a group of Turkish patients with Hashimoto’s thyroiditis.. Minerva Endocrinologica..

[30] Gao XR, Yu YG (2018). Meta-Analysis of the Association between Vitamin D Receptor Polymorphisms and the Risk of Autoimmune Thyroid Disease.. Int J Endocrinol..

